# P04.29. Tai chi and health related quality of life: a systematic review and meta-analysis of randomized controlled trials

**DOI:** 10.1186/1472-6882-12-S1-P299

**Published:** 2012-06-12

**Authors:** S Abariga, C Wang

**Affiliations:** 1 Tufts Medical Center, Division of Rheumatology, Boston, USA

## Purpose

Tai Chi, a Chinese mind-body exercise, has a complex multicomponent therapy that integrates physical, psychosocial, emotional and behavioral elements to promote health in a variety chronic condition. Health Related Quality of Life (HRQL) is a multidimensional, subjective patient centered outcome that encompasses those conditions that affects a person’s overall well-being. A comprehensive review of the literature of Tai Chi and quality of life is an important step for understanding the mind-body beneficial effects. We conducted a systematic review and meta-analysis of randomized controlled trials evaluating the effect Tai Chi on HRQL on a variety of populations.

## Methods

We performed a comprehensive search of 11 data databases through October 2011 with no language restriction. We included randomized controlled trials evaluating Tai Chi in HRQL for both healthy and patients with chronic conditions with a sample size of least 10 subjects; at least two weeks of follow-up and assessed HRQL as an outcome. Study quality was assessed with the Jadad instrument. The differences between treatment groups were reported as mean change (95% CI, p-value). We also conducted a meta-analysis on studies using the SF-36 quality of life instruments.

## Results

We identified 61 potentially relevant studies and 31 RCTs with a total of 2662 subjects met our eligibility criteria. Of these, six RCTs used SF-36 and 25 used other HRQL measures. Twenty-six of them reported an improvement in HRQL with Tai Chi practice compared with control. The meta-analysis results showed that five RCTs, average quality of four, enrolling 231 patients with 12 to 15 weeks of Tai Chi therapy, had benefit effects. The pooled effect size for the physical component score was 6.08 (95% CI: 3.7 to 8.45) and for the mental component score was 4.12 (95% CI: 1.32 to 6.93).

## Conclusion

The current body of evidence suggests that Tai Chi may improve HRQL across various disease populations.

